# Resolving “orphaned” non-specific structures using machine learning and natural language processing methods

**DOI:** 10.3897/BDJ.6.e26659

**Published:** 2018-08-10

**Authors:** Dongfang Xu, Steven S Chong, Thomas Rodenhausen, Hong Cui

**Affiliations:** 1 University of Arizona, Tucson, United States of America University of Arizona Tucson United States of America; 2 National Center for Ecological Analysis and Synthesis, University of California, Santa Barbara, United States of America National Center for Ecological Analysis and Synthesis, University of California Santa Barbara United States of America

**Keywords:** Information Extraction, Machine Learning, Anaphora Resolution, Ontology Application, Biodiversity Literature, Morphological Descriptions, Performance Evaluation

## Abstract

Scholarly publications of biodiversity literature contain a vast amount of information in human readable format. The detailed morphological descriptions in these publications contain rich information that can be extracted to facilitate analysis and computational biology research. However, the idiosyncrasies of morphological descriptions still pose a number of challenges to machines. In this work, we demonstrate the use of two different approaches to resolve meronym (i.e. part-of) relations between anatomical parts and their anchor organs, including a syntactic rule-based approach and a SVM-based (support vector machine) method. Both methods made use of domain ontologies. We compared the two approaches with two other baseline methods and the evaluation results show the syntactic methods (92.1% F1 score) outperformed the SVM methods (80.7% F1 score) and the part-of ontologies were valuable knowledge sources for the task. It is notable that the mistakes made by the two approaches rarely overlapped. Additional tests will be conducted on the development version of the Explorer of Taxon Concepts toolkit before we make the functionality publicly available. Meanwhile, we will further investigate and leverage the complementary nature of the two approaches to further drive down the error rate, as in practical application, even a 1% error rate could lead to hundreds of errors.

## Background

Using the large volumes of information contained in biodiversity literature, we aim to provide scientists with rich computable data so they can build a more complete tree of life, predict the causal genes for wider ranges of diseases/conditions, derive better models of the decline of species populations and improve climate change predictions. Biodiversity literature contains various descriptive information on extinct and extant taxa, including habitat, distribution, phenology, ecology, physiology and morphology. In this work, we are primarily concerned with making the information contained in morphological descriptions more accessible to machines. One way to accomplish this is to extract character information from descriptions into a structured format, such as taxon-by-character matrices. This work involves linking a structure/organ (e.g. *leaf* or *stem*) with its properties (e.g. *colour*, *shape*, *orientation* etc., often called “characters” in systematics biology), as described in the literature (Dececchi et al. 2015). While developing the Explorer of Taxon Concepts (ETC) toolkit web application (Cui et al. 2016), we encountered the challenge of resolving “orphaned” parts extracted by the ETC Text Capture tool. The orphaned parts occur because taxonomic descriptions often include a large set of terms, representing non-specific structural parts, such as base, apex and tip. In this paper, we refer to such terms as non-specific structure (NSS) terms. They are *non-specific* because these terms stand alone and do not correspond to any identifiable organ; instead, they could be part of a number of different organs. For example, *leaf*, *stem* and *petal *could each have an *apex*, therefore *apex *is a NSS. When a NSS is linked to its parent organ, as in* leaf apex*, it represents an independent entity and we say it is resolved. Extracting character information about such structures requires the machine to bridge the non-specific structures with their anchor organs. Following Poesio and Vieira’s work (Poesio and Vieira 1998), the term *anchor *is used as a generalisation of the term *antecedent* to indicate the discourse entity to which an anaphoric expression is related, regardless of its relative location. For example, associating *apex acuminate* with its appropriate organ, *leaf*, produces a complete and accurate character *leaf apex*accuminate.

In computational linguistics, this problem is treated as a subclass of the bridging reference problem, whose resolution requires not only finding the anchor in the text to which the bridging reference is related, but also identifying the relationship between them (Sindner 1979, Poesio and Vieira 1998, Clark 1975, Grosz 1977, Hawkins 1978, Fraurud 1990). Characterised by the relationship between the reference and the anchor, a bridging reference may be classified as an anaphoric reference or associative reference; in the former case, the reference and the anchor denote the same entity (e.g. Mr. Smith and he), while in the latter case the two entities are associated. In the case of associative references, further distinction can be made based on the relation, for example, synonyms, hypernyms, hyponyms or meronyms (Poesio and Vieira 1998). In our study, we focus on mereological bridging references because the relationships between bridging references and anchors in this case are part-of relationships. Resolving part-of relationships amongst anatomical structures is already needed to extract phenotypic characters from vast amounts of taxonomic descriptions continuously being published in human languages.

An illustrative example of our task is given below:

*Leaflets articulated, inserted near the edges of the* *rhachis** towards the* *adaxial side, lacking a differently coloured basal gland; stomata on lower surface only or on both surfaces; epidermal cells elongated parallel to long axes of leaflets.*

In this example, the goal is to associate *edges, adaxial side, lower surface, surfaces, cells *and* long axes* with their anchors (parents) *rhachis*, *leaflets*, *leaflets*, *leaflets***, ***epidermal*********and *leaflets*, respectively.

This paper reports the evaluation of two methods we developed to resolve NSS in taxonomic descriptions. We start by giving a formal task definition and then, in the Data Resources section, we describe the corpus, datasets and ontologies used to develop and evaluate the methods. After introducing the two methods, we present evaluation results. We conclude the paper with future directions for research.

## Task definition

Morphological descriptions of the biodiversity domain pose a number of challenges to existing natural language processing (NLP) algorithms. Thessen et al. (2012) identified three characteristics about morphological descriptions that make them difficult to parse using existing methods: 1) Specialised language is used and a large number of terms are not contained in pre-compiled lexica. Ambiguity is also more prevalent in biological text than texts from other domains (Chen et al. 2004). In addition, there is a heavy use of abbreviations in life science literature. 2) Morphological descriptions are very diverse amongst and within taxon corpora. 3) The syntax of morphological descriptions often differs from standard syntactic structure in English, i.e. a telegraphic sub-language that lacks verbs is used. The ETC toolkit (http://etc.cs.umb.edu/etcsite) is the only web-based, publicly accessible application to our knowledge that extracts character data from organismal morphological descriptions to support biological research. The toolkit has been used by biology research projects, for example, the extraction of phenotypic traits for the tree of life (Endara et al. 2018), the Building a Comprehensive Evolutionary History of Flagellate Plants project (US NSF DEB-1541506), the Botanical Knowledge Portal project (co-sponsored by Agriculture and Agri-Food Canada) and the Flora of North America project.

Although natural language processing tools and algorithms have been successfully implemented in various tasks, bridging references are considered one of the most challenging problems in discourse analysis since the knowledge about the events and natural objects required to identify each relation goes beyond the text itself (Clark 1975). While various work has been published on this subject (Poesio et al. 2004, Bunescu 2003, Fan et al. 2005), most of those efforts used traditional linguistic methods rather than machine-learning approaches. We are not aware of open source algorithms that resolve meronym associative reference problems in taxonomic descriptions. Our work represents a small but solid contribution to this area.

Given a short piece of text (one or a few sentences) from a morphological description that contains some NSS terms, our task was to resolve the NSSs, i.e. identify the anchor (parent) structure for each NSS. More formally, given a set of NSS-terms A = {a_1_, a_2_, a_3_,…, a_n_} and a text segment W = {w_1_, w_2_, w_3_, ..., w_n_} consisting of n tokens, for any given w_i_=a_j_ and, amongst all semantically valid part-of relations (w_i_, w_k_), our task was to find the specific w_k_ that would resolve w_i_ as the intended independent entity described by the description.

## Data resources

A total of 7562 sentences from 3876 morphological description files of 11 taxon groups were extracted from various taxonomic data sources we have collected in prior years, including Plazi.org and the Flora of North America (FNA). A list of 39 NSS terms pertinent to these descriptions was created by domain experts. Table 1 shows the NSS terms along with the number of occurrences in the corpus.

An initial dataset consisting of 169 sentences were randomly selected from taxonomic descriptions of ants, mushrooms and plants with 389 NSS term occurrences. This was used as the dataset to develop the two methods reported in this paper. To expand the taxon and NSS terms coverage, another 167 sentences were sampled in a stratified random manner from the corpus to form the test dataset; sentences were randomly selected from all 11 taxon groups and each sentence contained at least one NSS term. 366 NSS term occurrences were in the test dataset. Sentences containing target anchors were added if they were not sampled in the first round. After the data collection procedure, sentences that contained both NSS term(s) and target anchor(s) were treated as statements. The composition of the training and test datasets is shown in Table 2. Statements in the development dataset and test dataset did not overlap.

**Generation of the annotated evaluation datasets**

The evaluation datasets (development and test) needed to be annotated with NSSs and their anchor organs. This was done semi-automatically. The fine-grained morphological character extraction system ETC Text Capture tool (Cui et al. 2016) was used to annotate all structures (i.e. biological entities, e.g. *elytron*), characters (e.g. *shape, rounded*) and relationships, including preposition and verb phrases in the statements (e.g. *on primary stem*). Since the raw annotations did not resolve NSSs, the annotations contained NSS structures as independent biological entities without their anchor structures. Statements with raw annotations were the input for the algorithms.

**Figure 1.**
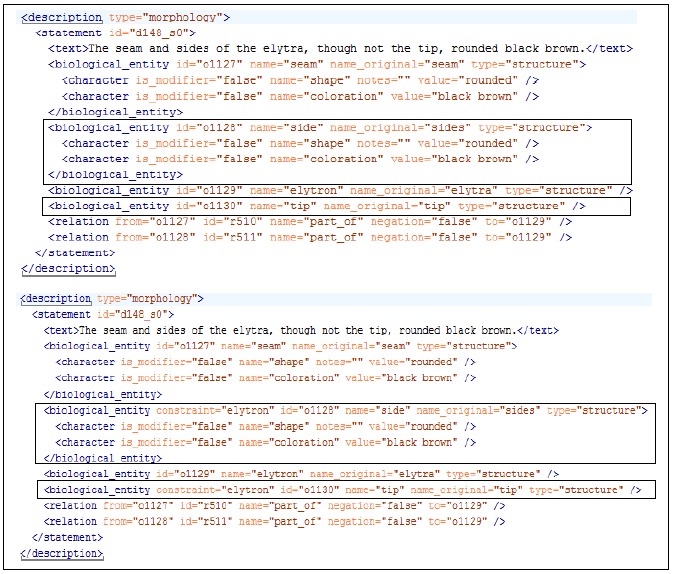
An example showing raw annotations (top) and gold standard annotations (bottom).

For performance evaluation, NSS-terms in both datasets were manually resolved by adding anchor organs to the NSS-terms. Fig. 1 shows an example of raw annotations and the annotated answer key (gold standard). The annotations were completed by one author and the gold standard was hidden from algorithm developers until the algorithms were ready for evaluation.

**The NSS Ontologies**

Anatomical and phenotype character ontologies are being created by bioinformatics communities to generate computable data and support integration of data silos (Köhler et al. 2016, Hoehndorf et al. 2016). Ontologies carry useful knowledge often not explicitly stated in morphological descriptions (Huang et al. 2015). To enable intelligent organisation and use of organism-based morphological information, ontologies need to be created on a per domain basis since research on different taxon groups employ different vocabularies and new terminology is constantly encountered (Cui 2010). In terms of non-specific structures resolution, different domains also use various non-specific structures. For example, in the domain of botany, *body* is a non-specific structure, while in insects or some single-cell organisms, *body* is a specific structure as there is only one body. Following previous research on domain ontology building (Chong et al. 2017), we used the Ontology Building tool in the ETC toolkit to create domain ontologies. The reason for creating task-specific ontologies, instead of using existing ontologies, such as Biological Spatial Ontology or Plant Ontology, is that existing formal ontologies often do not include non-specific structures. For part-of relationships to be included in a formal ontology, the related entities must meet the so-called 'all some' rules. To say *apex *is part of *leaf* in a formal ontology, all apices must be part of some leaves. However, this is not true for all cases (e.g. an *apex *could be a *stem apex*), so this relationship cannot be included in a formal ontology (e.g. the Plant Ontology). In general, non-specific structures are not included in OBO Foundry (Open Biological and Biomedical Ontology Foundry) ontologies for this reason. The ontologies we created in our task only specify that an *apex *could be part of a *leaf* and an *apex *could be part of a *stem*. The soft constraints in our ontologies express the potential of part-of relationships and the algorithms to act upon those possibilities and select the most likely parent structure.

In this experiment, the tool was used by the same author who subsequently annotated the answer keys to create ontologies holding part-of relationships amongst various biological structures for the development and test data, respectively. Taking the statement in Fig. 1 as an example, we used the tool to first add the structures *elytron*, *side***, ***tip***,***elytron side*****and*****elytron tip *into the ontologies and then linked the terms with part-of and subclass relationships (e.g. *elytron side *is a subclass of*****side* and a****part-of*****elytron*). A two-month gap separated each of the ontology building events and gold standard annotation to control for any potential confounding between the ontologies and the gold standard. A total of 488/540 unique structures (including organ and NSS-terms) and 392/351 part-of relationships were created in the development/test versions of the NSS ontologies.

## Methods

### Syntactic rule method

This method took advantage of the syntactic parsing results embedded in the raw annotation of the input and utilised a set of simple rules to resolve the NSSs (i.e. finding the anchor organ for a NSS). Four rules were used: (1) biological entity terms within three-sentence boundaries of a NSS were considered candidate anchor organs (i.e. window size = 3); (2) part-of relationships generated by the ETC Text Capture tool through the of-phrases containing a NSS term, such as “*baseof the leaves*”; (3) possession words around a NSS-term, such as “with”, “contain” and “have”; (4) the NSS ontologies.

Method 1 shows the pseudocode to obtain the mereological bridging references for NSS terms in each statement. First, it defined a queue to store all candidate anchor terms (i.e. biological entities, including all structure terms extracted by ETC) marked in one statement. It then sorted the NSS terms based on the order of their appearance in the queue and stored them into a list. For each NSS term in the list, it initialised a stack and pushed the current NSS term to the stack_NSS-to-be-resolved. The algorithm then used each rule in the list of rules [(2), (3), (1)] to find the candidate anchor term that satisfied one of the above rules (see #9 in pseudocode) and appeared in the ontology (#12). The list of rules [(2), (3), (1)] was ordered by their confidences (i.e. the order of confidences in rules for our task were: of-phrase > possession words > window size). Specifically, the algorithm first found any candidate anchor term in queue_terms that satistified both the of-phrase rule (2) and the ontology rule (4). If either rule was not satisfied, it continued checking rules (3) and (4) and then rules (1) and (4). The NSS-term was resolved if the above procedures returned any candidate anchor term, otherwise a null value was returned. 

We also implemented a recursive approach to resolve bridging reference cases where multiple NSS terms were involved, such as “abdomen has a thin edge at its upper margin”; here, the anchor term of “edge” is “upper margin” which itself is also a NSS. To handle such situations, before the algorithm returns the candidate anchor term, it checks whether the candidate term is one of the NSS terms. If the answer is true, the algorithm then saves the relationship of the current NSS term and the candidate term (the new NSS term) and recursively finds the anchor term for the new NSS term until the new candidate anchor term is determined to be not a NSS. For the above example, the algorithm found the anchor term of “edge” to be "upper margin", which is also a NSS term. The algorithm then pushed the "upper margin" into the stack_NSS-to-be-resolved and resolved the new NSS term "upper margin". After finding the anchor term of "upper margin" to be "abdomen", the algorithm updated the anchor term of "edge" to be "abdomen upper margin".

#1 Initialize NSS Ontology, queue_terms, list_NSS-terms

#2 for entity in sentence do //store all biological entities: #2-#4

#3 insert entity into queue_terms

#4 end for

#5 list_NSS-terms = get_NSS(queue_terms) //obtain all NSS mentions

#6 for each NSS-term in list_NSS-terms do //resolve NSS term: #6-#28

#7 initialise stack_NSS-to-be-resolved

#8 push NSS-term to stack_NSS-to-be-resolved 

#9 for each rule in rules [(2), (3), (1)] do

#10 for term in queue_terms do

#11 if rule(term, NSS-term) then //rules for terms: #11-#25

#12 if (term, NSS-term) in Ontology then

#13 if term is not in NSS-terms then//recursion: #13-23 

#14 while stack_NSS-to-be-resolved is not empty do

#15 NSS-term ← pop(stack_NSS-to-be-resolved)

#16 anchor (NSS-term) ← term

#17 in list_NSS-terms delete NSS-term

#18 term ← term + NSS-term 

#19 else:

#20 push term to stack_NSS-to-be-resolved

#21 NSS-term ← term

#22 goto #9

#23 end if 

#24 end if

#25 end if

#26 end for

#27 end for

#28 end for

Method 1 Mereological Bridging Reference using Syntactic Rules and the NSS Ontologies.

This method and one variation of it were eventually evaluated. The variation resolves a NSS using a NSS ontology and the constraint of window size=3 (rules 1 and 4) and ignores the part-of and possession clues (rules 2 and 3). The source code for the Syntactic Method can be found at https://github.com/biosemantics/charaparser/tree/master/enhance.

### Support vector machine

Support vector machine (SVM) functioning as a classifier remains an effective, low cost and robust method for many NLP tasks, especially for problems with a small number of training instances (Zeng et al. 2014, Moraes et al. 2013).  We followed a common relation extraction strategy and framed the mereological bridging reference resolution as a pairwise relation classification problem. Our classifier used three groups of features:

1. Distance and position features: the indicator of whether the anchor term was the subject of a sentence/clause; the indicator of whether the anchor term was the closest term to the NSS term; absolute distance between the two structure terms in a statement; relative distance between the two structure terms in a statement (i.e. the absolute distance divided by the number of tokens in a statement); the absolute number of structure term(s) between the two structure terms; the relative number of structure term(s) between the two structure terms (i.e. the absolute number divided by the number of structures in a statement).
2. Bag-of-word features: the indicators of whether the connectors between the term pairs were in the set of “in, on, at, of, has, have, with, contains, without”; tokens themselves before and after both structure terms in a certain context window, excluding the connectors listed above.
3. Semantic features: the indicator of whether the term pairs appeared in the NSS ontology; the indicator of whether the anchor term was a NSS.

We used the LIBSVM implementation (Chang and Lin 2012) to train a SVM classifier to output probability estimates for each NSS-term and anchor term pair. The predicted anchor term for each NSS term, i.e. \begin{varwidth}{50in}
        \begin{equation*}
            anchor\_term(NSS \,term)
        \end{equation*}
    \end{varwidth} was selected from the pair which had the maximum probability amongst all term pairs, formally defined below:

\begin{varwidth}{50in}
        \begin{equation*}
            anchor\_term(NSS \,term)= argmax_{x &amp;#x02208;X} \, Pro(x,NSS \,term)
        \end{equation*}
    \end{varwidth}

where \begin{varwidth}{50in}
        \begin{equation*}
            X
        \end{equation*}
    \end{varwidth} is the set of biological entity terms within one statement, excluding the NSS term which is to be resolved and \begin{varwidth}{50in}
        \begin{equation*}
            Pro(x,NSS \,term)
        \end{equation*}
    \end{varwidth} is a probability function which calculates the probability of \begin{varwidth}{50in}
        \begin{equation*}
            x
        \end{equation*}
    \end{varwidth} being the anchor term of the \begin{varwidth}{50in}
        \begin{equation*}
            NSS \,term
        \end{equation*}
    \end{varwidth}. We used 5-fold cross validations to adjust the following parameters for the best performance: the word frequency threshold was set to 9 and the context window to 4 for the bag-of-word features; class-weight was set as 7 to handle the unbalanced classes; we experimented with multiple kernels and selected the polynomial kernel function with the degree set to 3; other parameters were set to their default.

This method and one variation of it were evaluated. The variation used features from groups 1 and 2, leaving out the semantic features. The source code of the SVM method can be found at https://github.com/biosemantics/SVM-for-Nonspecific-Structure.

### Two baselines: subject entity and closest entity

Two additional baseline algorithms were also implemented and evaluated. For each NSS term, the first baseline algorithm (Baseline 1) always chose the subject entity term (i.e. the first entity appearing in a statement) as its anchor term, while the second baseline algorithm (Baseline 2) always identified the closest entity term around the NSS term as its anchor term. Taking the example from the Background section, NSS term *edges* would be linked to *leaflets *using the Baseline 1 algorithm or linked to* rhachis* using the Baseline 2 algorithm. 

### Evaluation metrics

The four methods and two variations were evaluated using precision, recall and f1 metrics, which are routinely used in information retrieval and information extraction tasks. Precision (P), recall (R) and f1 (F1) are formally defined below:

\begin{varwidth}{50in}
        \begin{equation*}
            P(S, H) = \frac {|S &amp;#x02229; H|} {|S| }
        \end{equation*}
    \end{varwidth}

\begin{varwidth}{50in}
        \begin{equation*}
            R(S, H) = \frac {|S &amp;#x02229; H|} {|H|}
        \end{equation*}
    \end{varwidth}

\begin{varwidth}{50in}
        \begin{equation*}
             F1(S, H) = \frac {2 * P(S,H) *R(S,H)} { P(S,H) + R(S,H) }
        \end{equation*}
    \end{varwidth}

where S is the set of anchor terms identified by the system and H is the set of anchor terms annotated in the gold standard.

## Results and discussion

We compared the different methods described in the Methods section. Table 3 shows the best performance results using the development dataset and the final results using the test dataset. First, it is evident that naive approaches such as the baseline methods were not sufficient to solve the problem, as they achieved F1 scores of 42.3% (Baseline 1) and 33.2% (Baseline 2).

Second, we found the syntactic approach outperformed the SVM method by a large margin. For example, the best syntactic approach achieved an F1 score of 92.1%, while the best SVM model achieved an F1 of only 80.7%. This may be due to the small training size provided to the SVM. From a practical point of view, it was not realistic to request that domain scientists annotate large sets of training examples. In contrast, they are more likely to construct an ontology that can be reused to process future descriptions and solve other related problems, such as extracting phenotypes.

Third, we found ontologies were reliable knowledge sources in resolving orphaned parts in morphological descriptions. Using an ontology alone, the syntactic rule-based approach achieved an F1 score of 91.4%. Using an ontology also improved SVM performance by 20 percentage points for the F1 score. The syntactic rules (of-phrases and possession words) seem to be useful for the rule-based method, but using these rules with an ontology only improved the F1 score by 0.7% and, when considering the 366 test examples, their effects on reference resolution were unclear since the results may have been confounded by usage of an ontology. We are planning to evaluate a second variation of the rule-based method using only the rules and not an ontology to further examine the effects of these rules.

**Figure 2.**
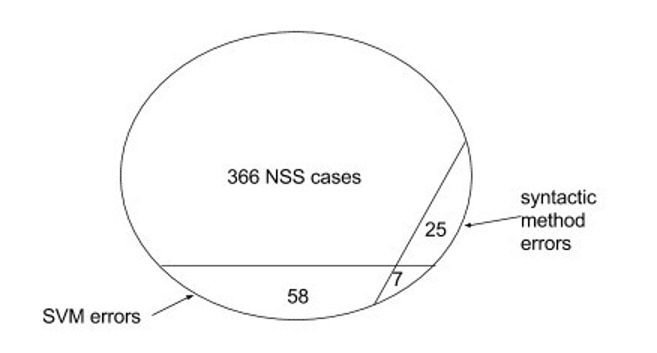
Error distribution between the best performances of the SVM and syntactic methods.

Our error analysis showed that the syntactic method and the SVM method were complementary to each other, as the mistakes made by the two methods were largely disjoint. Fig. 2 shows the error distribution between the best performances of the SVM and syntactic methods. Amongst 366 NSS terms to be resolved, the SVM method made 65 mistakes while the syntactic method made 32 mistakes, but they were both incorrect in only 7 of the same cases. 

## Conclusions and future work

With the results from the dataset covering a wide range of descriptions, we can tentatively conclude that the syntactic rule method is what the ETC toolkit will adopt, given that ETC toolkit already provides a user-friendly ontology building tool for domain scientists and students to create ontologies. However, in the immediate future, it is worthwhile for additional testing on the development version of ETC toolkit before we make the functionality publicly available and to further investigate and leverage the complementary nature of the syntactic rule method and the SVM method to minimise the error rate, as in practical application NSS, terms are commonly used in morphological descriptions and even a 1% error rate could lead to numerous errors.

## Figures and Tables

**Table 1.** Non-specific structure terms and their occurrence in the corpus.

**NSS term**	**count**	**NSS term**	**count**	**NSS term**	**count**	**NSS term**	**count**
apex	511	centre	52	groove	350	protuberance	8
appendix	13	chamber	14	layer	25	remnant	28
area	476	component	7	line	163	section	112
band	114	concavity	2	margin	2419	side	609
base	684	content	22	middle	271	stratum	2
belt	2	crack	8	notch	80	surface	959
body	1171	edge	258	part	264	tip	82
cavity	148	element	63	pore	101	wall	23
cell	120	end	166	portion	240	zone	51
centre	49	face	727	projection	37		

**Table 2.** Development and test datasets composition.

**Taxon**	**Total Number of Sentences**	**Development Dataset** **(# of statements)**	**Test Dataset** **(# of statements)**
Ants	866	21	20
Bees	2189	18	21
Birds	516	13	10
Diatoms	503	13	18
Ferns	630	15	7
FNA v-5 (pink, leadwort and buckwheat families)	686	13	9
Gymnodinia	206	10	13
Mushrooms	815	26	35
Nematodes	604	15	18
Sponges	92	13	5
Weevils	455	12	11
Total	7562	169	167

**Table 3.** Performances of the Baseline, Syntactic and SVM Methods Using the Development and Test Datasets.

**Methods**	**F1 (Development)**	**P (Test)**	**R (Test)**	**F1 (Test)**
Baseline 1 (subject entity)	63.9%	42.3%	42.3%	42.3%
Baseline 2 (closest entity)	30.3%	33.2%	33.2%	33.2%
Syntactic (ontology only)	91.1%	92.2%	90.5%	91.4%
Syntactic (all rules)	93.7%	93.0%	91.3%	92.1%
SVM (feature groups 1 and 2)	76.1%	60.9%	60.9%	60.9%
SVM (all features)	89.6%	80.7%	80.7%	80.7%
